# Formaldehyde-Induced Aggravation of Pruritus and Dermatitis Is Associated with the Elevated Expression of Th1 Cytokines in a Rat Model of Atopic Dermatitis

**DOI:** 10.1371/journal.pone.0168466

**Published:** 2016-12-22

**Authors:** Rafael Taeho Han, Seung Keun Back, Hyunkyoung Lee, JaeHee Lee, Hye young Kim, Hee Jin Kim, Heung Sik Na

**Affiliations:** 1 Neuroscience Research Institute & Department of Physiology, Korea University College of Medicine, Seoul, Korea; 2 Department of Pharmaceutics and Biotechnology, College of Medical Engineering, Konyang University, Chungnam, Korea; 3 Division of Biological Science and Technology, Science and Technology College, Yonsei University Wonju Campus, Wonju, Korea; Gentofte Hospital, DENMARK

## Abstract

Atopic dermatitis is a complex disease of heterogeneous pathogenesis, in particular, genetic predisposition, environmental triggers, and their interactions. Indoor air pollution, increasing with urbanization, plays a role as environmental risk factor in the development of AD. However, we still lack a detailed picture of the role of air pollution in the development of the disease. Here, we examined the effect of formaldehyde (FA) exposure on the manifestation of atopic dermatitis and the underlying molecular mechanism in naive rats and in a rat model of atopic dermatitis (AD) produced by neonatal capsaicin treatment. The AD and naive rats were exposed to 0.8 ppm FA, 1.2 ppm FA, or fresh air (Air) for 6 weeks (2 hours/day and 5 days/week). So, six groups, namely the 1.2 FA-AD, 0.8 FA-AD, Air-AD, 1.2 FA-naive, 0.8 FA-naive and Air-naive groups, were established. Pruritus and dermatitis, two major symptoms of atopic dermatitis, were evaluated every week for 6 weeks. After that, samples of the blood, the skin and the thymus were collected from the 1.2 FA-AD, the Air-AD, the 1.2 FA-naive and the Air-naive groups. Serum IgE levels were quantified with ELISA, and mRNA expression levels of inflammatory cytokines from extracts of the skin and the thymus were calculated with qRT-PCR. The dermatitis and pruritus significantly worsened in 1.2 FA-AD group, but not in 0.8 FA-AD, compared to the Air-AD animals, whereas FA didn't induce any symptoms in naive rats. Consistently, the levels of serum IgE were significantly higher in 1.2 FA-AD than in air-AD, however, there was no significant difference following FA exposure in naive animals. In the skin, mRNA expression levels of Th1 cytokines such as TNF-α and IL-1β were significantly higher in the 1.2 FA-AD rats compared to the air-AD rats, whereas mRNA expression levels of Th2 cytokines (IL-4, IL-5, IL-13), IL-17A and TSLP were significantly higher in 1.2 FA-naive group than in the Air-naive group. These results suggested that 1.2 ppm of FA penetrated the injured skin barrier, and exacerbated Th1 responses and serum IgE level in the AD rats so that dermatitis and pruritus were aggravated, while the elevated expression of Th2 cytokines by 1.2 ppm of FA in naive rats was probably insufficient for clinical manifestation. In conclusion, in a rat model of atopic dermatitis, exposure to 1.2 ppm of FA aggravated pruritus and skin inflammation, which was associated with the elevated expression of Th1 cytokines.

## Introduction

Atopic dermatitis (AD) is a chronic relapsing inflammatory skin disease characterized by pruritus and eczema [1] and affects adults and children with worldwide prevalence rates of 1~20% [2]. The development of AD has been attributed to complex interactions of genetic predisposition and environmental factors such as immune dysregulation and exposure to surrounding allergens [3, 4]. A number of abnormalities play critical roles in the pathogenesis of AD including Th2-skewed cytokine expression in the lesions [5, 6], mutation in the filaggrin gene [7], and elevated serum IgE, which is probably correlated with the severity of the disease [8, 9]. Moreover, environmental triggers provoke a mixed immune response of Th1 and Th17 in the progression of AD and the resulting cytokine signaling elicited subsequent inflammation unrelated to the early Th2 response [6, 10, 11].

Of those environmental factors, not only allergens but also air pollution, which is increasing with urbanization, can potentially cause the progression and aggravation of AD. This is known as the "outside-inside" hypothesis [12]. Several lines of evidence suggest that biological allergens probably contribute to the pathophysiology of AD and the increasing level of chemical air pollution might affect the prevalence of allergic diseases [12–14]. Recent studies on the biological effects of indoor air pollution provided evidence that pollutants might be an aggravating factor for allergic diseases. However, the previous studies on air pollution in allergic diseases were mostly conducted from the epidemiology perspective [12, 15, 16], and were primarily focused on airway diseases such as asthma [17, 18], or aimed at examining the effect of cutaneous exposure to formaldehyde (FA), one of the most common and dangerous indoor air pollutants [19, 20], on naive animals [21, 22]. Thus, the precise role of air pollutants, in particular FA, in the development and/or aggravation of AD still remains elusive.

Recently, we developed a rat model of atopic dermatitis produced by neonatal capsaicin treatment. The model showed chronically relapsing dermatitis and scratching behaviors accompanied by increases in serum IgE level and skin Th2 responses [23].

In this study, using this model, we investigated whether the exposure to FA induced and aggravated dermatitis and pruritus associated with related changes, such as serum IgE level and Th1 & Th2 cytokines, in naive and AD rats, respectively.

## Materials & Methods

### Animals

All experiments were approved by the Korea University College of Medicine Animal Research Policies Committee. Pregnant Sprague-Dawley rats (Samtako) were obtained 2 or 3 days before parturition and delivered. Pups were weaned at postnatal day 21 and only the males were used in the present study. All animals were raised in a room maintained at a 12 h light/dark cycle (light on at 7:00 AM) at 22-%°C and had ad libitum access to food and water.

### A rat model of atopic dermatitis

Neonatal treatment with capsaicin (Sigma, 360376) caused atopic dermatitis-like symptoms in the rats. As previously described [23], the newborn pups were subcutaneously administrated with capsaicin of 5g/L (50 mg/kg) or vehicle (saline containing 10% Tween 80 and 10% ethanol) at a volume of 10 ml/g body weight within 24 h of birth.

### FA exposure

Paraformaldehyde (Merck, Germany) was diluted in distilled water and vaporized with a Medical Regulator MB-10 (Chiyoda Seiki, Japan). The vaporized gas was passed through a sealed chamber (40cm * 35cm * 60 cm) with two outlets for the input and output. The mean concentration of FA in the gas of 0.5% and 0.1% FA solution was 1.16±0.11 (1.2 ppm) and 0.83±0.01 (0.8 ppm), respectively, and the measurement was conducted by Seegene Medical Foundation, according to the manufacturer's protocol.

### Establishment of animal groups

Three days after birth, the AD and naive rats were exposed to 1.2 ppm of FA, 0.8 ppm of FA, or fresh air (Air) for 6 weeks (2 hours/day and 5 days/week). Six groups consisting of the 1.2 FA-AD, 0.8 FA-AD, Air-AD, 1.2 FA-naive, 0.8 FA-naive and Air-naive groups were established. The 1.2 FA-AD group was composed of 12 rats, the Air-AD group was composed of 10 rats, the 0.8 FA-AD, 1.2 FA-naive, and Air-naive groups were composed of 6 rats, and the 0.8 Air-naïve group was composed of 5 rats. Three litters were employed for the 1.2 FA-AD and Air-AD groups and two litters were used for the other groups.

### Assessment of itch

Rats were placed into separate plastic chambers (20 cm * 30 cm * 20 cm) equipped with a mirror to allow for a full coverage. After 30 minutes of habituation, the scratch behaviors of the rats were recorded for an hour using a digital video camera (HDR-CX380, Sony). The video clips were played back and the number of scratches was counted by blind experimenters who were unaware of which rats were assigned to which treatments. A bout of consecutive scratching strokes was considered one scratch (Video in S1 Video).

### Evaluation of skin lesion

Skin lesions were carefully assessed by scoring systems as previously described with modification [24]. The skin condition in three regions (the ears, face, and back) was evaluated every week for 6 weeks to determine the extent and severity of lesions. The unit size for the extent of skin lesions was 0.25cm^2^ and the dermatitis score was calculated by the severity index, which is defined in Table in S1 Table, multiplied by the unit size.

### Collection of the blood, the skin and the thymus

After 6 weeks of behavioral tests, 5~6 rats from each group were randomly selected and were sacrificed using CO_2_ gas and the blood in the right atrium was collected using a 1ml syringe with a 26-gauge needle. The obtained blood samples were centrifuged with 2500g in 4°C for 10 minutes. The supernatant was collected and the procedure repeated two more times. The lesional skin in the middle of the back of the neck (or the nearest lesional part) was excised and acquired in AD rats. The non-lesional skin in the same part of the body was excised and acquired in the naive rats. The left lower part of the thymus was collected. The samples were stored at -70°C and used within 2 weeks of collection.

### Enzyme linked immune sorbent assay (ELISA)

Serum IgE level was quantified using a rat IgE ELISA Kit (CUSABIO), according to the manufacturer's protocol.

### Quantitative RT–PCR

1mL of TRIzol Reagent (Life Technologies) was added per 50mg of tissue sample, which was homogenized using HG-15D (DAIHAN Scientific Co.) three times at 3000g for 10 seconds. RNA was extracted from the homogenized tissues according to the manufacturer's protocol. The extracted RNA samples were reverse-transcribed using M-MLV reverse transcriptase (Invitrogen) according to the manufacturer's protocol. The cDNA samples were amplified by quantitative PCR with a LigthCycler 96 (Roche Diagnostics) and the GoTaq qPCR Master Mix (Promega), according to the manufacturer’s instructions. The following primers were used: rat IL-1β (forward 5’-CTCCATGA-GCTTTGTACAAG-3’ and reverse 5’-TGCTGATGTACCAGTTGGGG-3’), IL-4 (forward 5’-ACCTTGCTGTCACCCTTC-3’ and reverse 5’-ACCTTGCTGTCACCCTGTT-C-3’), IL-5 (forward 5’-GAGGGGGCACTGTGGAAATA-3’ and reverse 5’-ACTCATCACGCCAAGGAACT-3’), IL-13 (forward 5’-CCTGGAATCCCT-GACCAACA-3’ and reverse 5’-GCCATAGCGGAAAAGTTGCT-3’), TNF-α (forward 5’-GAAAGTCAGCCTCCTCTCCG-3’ and reverse 5’-CTCCAAAG-TAGACCTGCCCG-3’), IL-17A (forward 5’-GTTCAGTGTGTCCAAACGCC-3’ and reverse 5’-AGGGTGAAGTGGAACGGTTG-3’), TSLP (forward 5’-TTCAATCCTGTCCCTGGCTG-3’ and reverse 5’-TGCAGGAAAGCCAC-AATCCTA-3’), GAPDH (forward 5’-ACTTTGGCATCGTGGAAGGG-3’ and reverse 5’-ACATTGGGGGTAGGAACACG-3’). Experiments were conducted in duplicate. Relative RNA expression level for each target gene was calculated using GAPDH as an internal control, which was previously tested in similar experimental conditions and properly validated [25, 26]. If the cycle number of relative RNA expression level of GAPDH was larger than 20, the samples were excluded from analysis. For analysis, we adopted difference between ΔCt of studied gene and control gene is calculated, then subtract between (so the value of the “ΔΔCt”) ΔCt of sample with unknown concentration and ΔCt of the calibrator. Normalized value of the expression level relative to the calibrator is determined by the Formula [27].

### Statistical analysis

**Fig 1:**
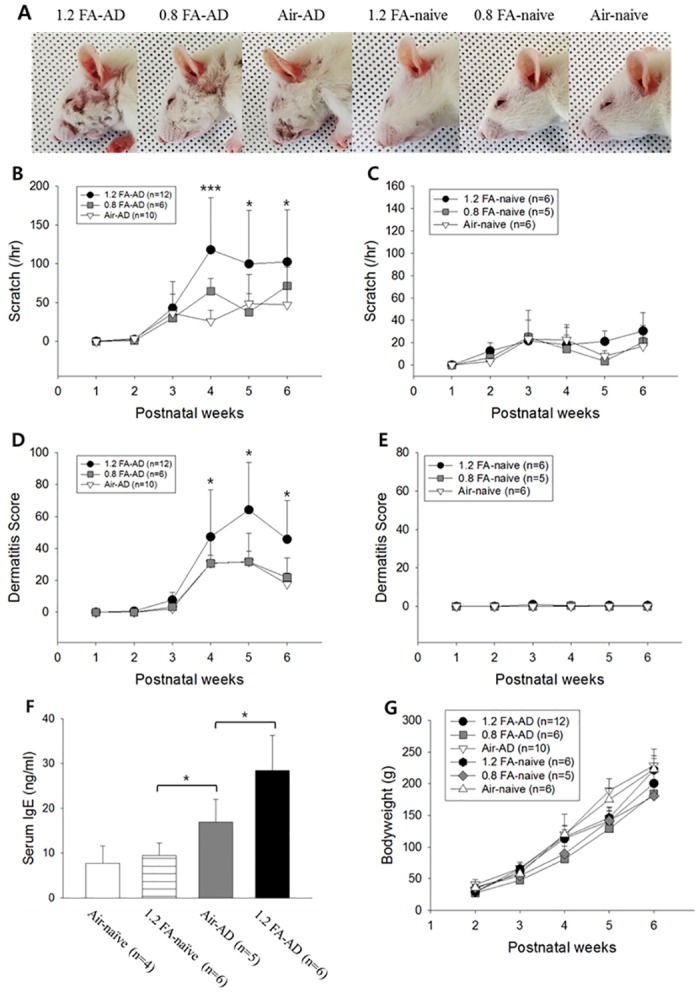
Aggravation of scratch and dermatitis, and increase in serum IgE level by FA exposure in AD rats. Photographs show dermatitis in the 5^th^ week. (A). The 1.2 FA-AD, but not 0.8 FA-AD, group shows significantly more severe scratch behavior (B) and dermatitis (D), compared to the Air-AD group. The 1.2 FA-AD group also shows significantly higher serum IgE level compared to the Air-AD group (F). However, there is no change in the symptoms (C & E) and serum IgE level (F) in naive groups. There is no significant difference in body weight among the six groups (G). Abbreviations; 1.2: 1.2 ppm, 0.8: 0.8 ppm, FA: formaldehyde, AD: atopic dermatitis, Air: fresh air. *p<0.05, ***p<0.001.

**Fig 2:**
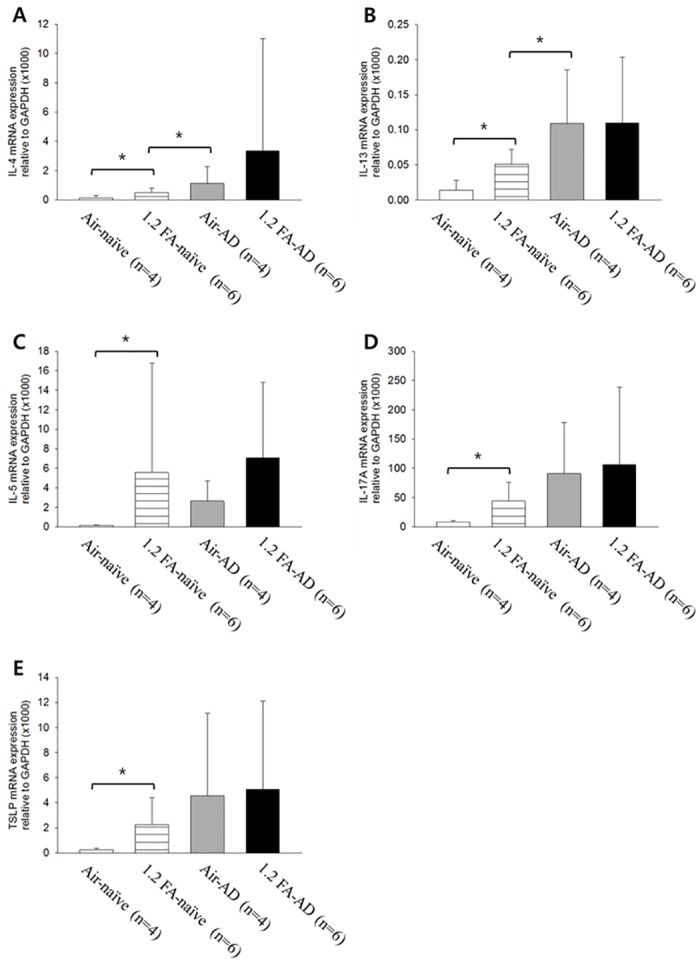
Elevation of expression of skin Th2 cytokines, IL-17A and TSLP by FA exposure in naive rats. The 1.2 FA-naive group shows significantly higher expression of Th2 cytokines such as IL-4, -5, and -13 (A~C), IL-17A (D) and TSLP (E) compared to the Air-naive group. However, the 1.2 FA-AD group does not show any significant difference in the expression level of Th2 cytokines (A~C), IL-17A (D) and TSLP (E) compared to the Air-AD group (A~E). Expression level of some of Th2 cytokines such as IL-4 (A) and IL-13 (B) is significantly higher in the Air-AD group than in the FA-naive group. Abbreviations; 1.2: 1.2 ppm, FA: formaldehyde, AD: atopic dermatitis, Air: fresh air. *p<0.05.

**Fig 3:**
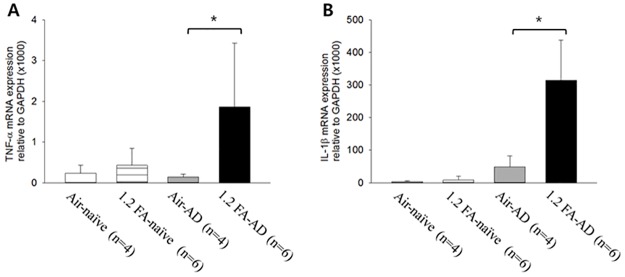
Elevation of expression of skin Th1 cytokines by FA exposure in AD rats. The 1.2 FA-AD group shows significantly higher expression of skin Th1 cytokines such as TNF-α (A) and IL-1β (B) compared to the Air-AD group. In contrast, there is no significant change in Th1 cytokines in naive rats. Abbreviations; 1.2: 1.2 ppm, FA: formaldehyde, AD: atopic dermatitis, Air: fresh air. *p<0.05.

**Fig 4:**
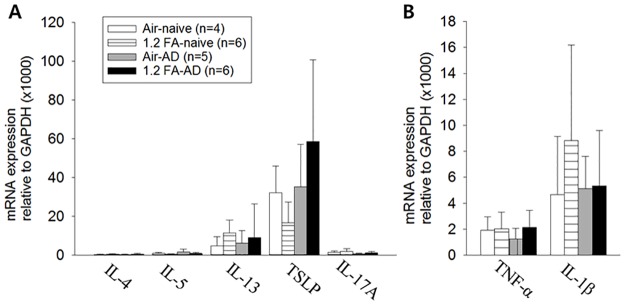
No change in expression of Th2 and Th1 cytokines by FA exposure in the thymus. No significant change in Th2 cytokines such as IL-4, IL-5 and IL-13, TSLP, IL-17A (A), and Th1 cytokines including TNF-α and IL-1β (B) in the thymus following exposure to 1.2 ppm of FA in both AD and naive rats. Abbreviations; 1.2: 1.2 ppm, FA: formaldehyde, AD: atopic dermatitis, Air: fresh air.

Results were expressed as means ± SEM. Data in Fig 1B–1E were analyzed using the Student’s t-test or the Mann–Whitney rank-sum test, depending on results from the normality test. Data in Fig 1F & 1G, in Figs 2, 3 and 4 were analyzed using one-way ANOVA followed by Tukey’s test or Kruskal-Willis test, depending on results from normality test. Statistical analysis was performed using SigmaStat 3.5 (Systat Software).

## Results

### Aggravation of dermatitis and pruritus by FA exposure

To confirm the effect of exposure to FA on atopic dermatitis, scratch behavior and dermatitis scores were assessed every week in the 0.8 FA-AD, 1.2 FA-AD, Air-AD, 0.8 FA-naive, 1.2 FA-naive and Air-naive groups for 6 weeks. The number of scratches and the dermatitis scores were significantly higher in the 1.2 FA-AD, but not the 0.8 FA-AD group compared to the Air-AD group (Fig 1A, 1B & 1D). On the other hand, there was no change in naive animals (Fig 1A, 1C & 1E). These results suggest that 1.2 ppm of FA aggravates dermatitis and pruritus in AD animals, but doesn’t induce symptoms in naive rats. However, in the respect of histological analysis, FA exposure doesn’t affect either inflammatory skin lesions from AD animals or naïve animals (S1 Fig).

Consistently, serum IgE level was significantly higher in the 1.2 FA-AD group compared to the Air-AD group (Fig 1F), while the 1.2 FA-naive group did not show a significant difference in serum IgE level compared to the Air-naive group. Additionally, the Air-AD group showed a significantly higher level of serum IgE compared to the 1.2 FA-naive rats (Fig 1F). These results suggest that 1.2 ppm of FA increases serum IgE level in AD animals, but not in naive rats, similar to dermatitis and pruritus.

To examine whether long-term exposure to FA causes growth retardation, we measured the body weight of all animals from 2 to 6 weeks after birth. The results showed that there was no significant difference in body weight among the six groups (Fig 1G). These results indicate that exposure to 1.2 ppm of FA does not induce growth retardation.

### Increase in skin Th2 cytokines, IL-17A and TSLP by FA exposure in naive rats

To elucidate whether the aggravation of dermatitis and pruritus induced by FA exposure is related to the changes in the skin Th2 cytokines, such as IL-4, -5, and -13 as well as IL-17A and TSLP, we examined whether exposure to 1.2 ppm of FA increased the expression of IL-4, IL-5, IL-13, IL-17A and TSLP in AD and naive rats. Intriguingly, the 1.2 FA-AD group did not show any significant differences in IL-4, IL-5, IL-13, IL-17A and TSLP compared to the Air-AD group (Fig 2A–2E). Additionally, IL-4 and IL-13 were higher in the Air-AD group than in the 1.2 FA-naive group (Fig 2A & 2B), similar to the results for serum IgE level (Fig 1F). These results imply that IL-4, IL-5, IL-13, IL-17A and TSLP do not play a key role in the FA-induced aggravation of dermatitis or pruritus in the rat model of AD. On the contrary, the 1.2 FA-naive group showed significant increases in IL-4, IL-5, IL-13, IL-17A and TSLP compared to the Air-naive group. These results imply that these increases are insufficient for the induction of dermatitis or pruritus in naive rats, given that the 1.2 FA-naive group does not show any symptoms.

### Increase in expression of skin Th1 cytokines by FA exposure in AD rats

To elucidate whether the aggravation of dermatitis and pruritus induced by FA exposure is related to the changes in skin Th1 cytokines, we examined whether exposure to 1.2 ppm of FA increased the expression of skin TNF-α and IL-1β in AD and naive rats. The 1.2 FA-AD group showed significant increases in TNF-α and IL-1β compared to the Air-AD group, but this increases was not observed in naive animals (Fig 3A & 3B), suggesting that TNF-α and IL-1β play a key role in the aggravation of dermatitis and pruritus induced by FA.

### No change in cytokine expression in the thymus by FA exposure

To examine whether the elevated expression of cytokines by FA exposure is restricted to the skin or prevailed in the internal organs, we analyzed the cytokine expression profiles of the thymus, which is a major site of immune regulation in rodents. No significant change in the expression of Th2 cytokines, TSLP and IL-17A (Fig 4A), and Th1 cytokines (Fig 4B) was observed in both of the AD and naive rats.

## Discussion

The results demonstrated that exposure to 1.2 ppm of FA exacerbated two major symptoms of atopic dermatitis, pruritus and dermatitis, in AD rats, but did not induce symptoms in naive rats. We also showed that aggravated dermatitis and pruritus in the AD rats were related to the elevated expression of Th1 cytokines, such as TNF-α and IL-1β, which are known to be associated with chronic AD lesions [10]. The aggravation was not related to Th2 cytokines, reported as crucial for the pathogenesis of atopic dermatitis [5].

Epidemiological studies [12, 15, 16] demonstrated that FA, one of the most common and dangerous indoor pollutant [19], contribute to the development of atopic dermatitis in healthy individuals as well as to the exacerbation of symptoms in patients with AD. However, in this study, although FA exposure worsened the symptoms in AD rats, it did not lead to the development of atopic dermatitis in naive rats. The discrepancy between the previous and present results was likely due to two possible scenarios. First, exposure to 1.2 ppm of FA in the present study was insufficient to initiate the development of the disease in naive rats, which was supported by our findings that the serum IgE and Th2 cytokine expression levels of the 1.2 FA-naive rats were lower than those of the Air-AD rats. Alternatively, we cannot rule out the possibility that the healthy subjects in the previous report had predisposing factors for atopic dermatitis, which contributed to the provocation of the FA-induced symptoms.

If, in AD rats, allergic reactions to FA played a role in aggravation of pruritus or dermatitis, naïve rats also underwent the provocation of the disease as AD rats. Furthermore, up to 2 ppm, gaseous FA exposure failed to produce to significant changes in human [28, 29]. Thus, it was less likely that aggravation of the diseases was attributed to simultaneous allergic contact dermatitis in AD rats.

Serum IgE level, which is correlated with the severity of the disease [8, 9], was increased by FA exposure in AD rats only. In agreement with our results, not only had Tarkowski et al [30] reported that serum IgE level was increased in sensitized mice following exposure to 2mg/m^3^, but Fujimaki et al. also had noted that FA exposure induced no differences in the serum IgE levels of naive animals [18].

According to Occupational Safety and Health Administration Fact Sheet, time weighted average and short-term exposure limits for FA is 0.75 ppm and 2.0 ppm, respectively. We used 1.2 ppm and 0.8 ppm of FA concentrations, which didn’t affect bodyweight of the rats (Fig 1G). In fact, we found that 1.8 ppm of FA was such a high concentration that caused growth retardation (data not shown). As shown in Fig 1B–1E, 0.8 ppm of FA didn’t affect the progression of the disease or induce symptoms in AD rats or naïve rats, respectively. Thus, we investigated the effect of 1.2 ppm of FA on the expression pattern of cytokines and serum IgE level in both AD rats and naïve rats.

Recent publications indicate that maternal separation in the early days induces alterations in behavior [31, 32]. The 0.8 FA-AD, 0.8 FA-naive and 1.2 FA-naive groups, which suffered from repetitive maternal separation for 2 hours a day similar to the 1.2 FA-AD group, showed few signs of behavioral alterations or growth retardation compared to the Air-AD or Air-naive groups. Thus, the aggravation of symptoms in the 1.2 FA-AD group is not likely to be caused by repetitive maternal separation.

As previously described, several lines of evidence demonstrated that Th2 cytokines, especially IL-4, 5, 10 and 13, were elevated in the skin of patients with AD [33, 34]. Several publications also demonstrated the importance of IL-17A and TSLP, which are associated with the activation of Th17 and the promotion of Th2 differentiation, respectively, in the development of the disease [35, 36]. In the present study, the results showed that AD rats did not exhibit overexpression of Th2 cytokines, IL-17A and TSLP following exposure to 1.2 ppm of FA. Rather, Th1 cytokines such as TNF-α and IL-1β were overexpressed. These results indicate that skin lesions in AD rats at 6 weeks are in the chronic status, in which Th1 cytokines play a more dominant role than Th2 cytokines [10]. FA exposure-induced aggravation of atopic dermatitis in AD rats is attributed to the increment of pro-inflammatory response [37].

In contrast, naive rats showed overexpression of Th2 cytokines such as IL-4, 5, and 13 following FA exposure in the present study. This is in line with prior studies, which showed that FA exposure mainly elevated Th2 cytokines in naive rats [38]. Although FA exposure did not provoke atopic dermatitis in naive rats, overexpression of Th2 cytokines such as IL-4, IL-5 and IL-13, and IL-17A and TSLP in the skin could provide a possible explanation for the potential contribution of FA exposure to the development of atopic dermatitis described in epidemiological studies [12].

To confirm whether FA exposure alters the expression of cytokines in other internal organs as well as the skin, we examined the cytokine expression profile of the thymus, which is one of the major immune regulatory organs in rodents [39]. Consistent with the previous results from Jung et al. [17], no significant alteration was observed in the present study.

There is a possibility of sensing the smell of formaldehyde. However, according to the reports [28, 29, 40], formaldehyde alone didn’t produce respiratory symptoms or induce IgE production in normal control. Thus, though we can’t exclude the possibility of the contribution of formaldehyde to allergy reaction via nasal mucosa or respiratory system, it might be less likely that our results were attributed to the formaldehyde allergy via respiratory systems. Furthermore, simple contact allergy to formaldehyde also possibly contributed to the aggravation of dermatitis and pruritus in our rat model of AD. However, if so, there must have been allergy reactions not only in model animals but also in naïve animals. The results showed that only model animals aggravated pruritus and dermatitis. Thus, it might be less likely that the aggravation was attributed to simple contact allergy to formaldehyde. In addition, according to the clinical study [28], up to 2 ppm, FA exposure failed to produce respiratory symptoms or significant changes in pulmonary function.

In further study, we need to examine whether FA exposure increases Th1-related inflammation from the histological point of view and whether the observation that Th1-skewed response contributes to FA induced-impairment of atopic dermatitis is replicable in other models of AD.

In conclusion, in AD rats, exposure to 1.2 ppm of FA contributed to the exacerbation of atopic dermatitis, which was related to the overexpression of Th1 cytokines and serum IgE level. On the contrary, although exposure to 1.2 ppm of FA increased Th2 cytokines, IL-17A and TSLP in naive rats, the effect seemed to be at the subclinical level.

## Supporting Information

S1 Video Video clip showing exemplary scratching strokes of a rat. (MP4)

S1 Table Modified severity index of dermatitis in a rat. (XLSX)

S1 Fig No change in the skin lesion between 1.2FA-AD and Air-AD, and between 1.2FA-naïve and Air-naïve in the aspect of histological analysis. No significant difference was observed in representative presentation from the skin lesion of 1.2FA-AD rat (A) and the Air-AD rat (B). No significant alteration was observed in representative presentation from the skin lesion of 1.2FA-naive rat (C) and the Air-naive rat (D).
(TIF)
