# Supplementary figures and images for: Formaldehyde-Induced Aggravation of Pruritus and Dermatitis Is Associated with the Elevated Expression of Th1 Cytokines in a Rat Model of Atopic Dermatitis
Source: PLoS One. 2016 Dec 22;11(12):e0168466. doi: 10.1371/journal.pone.0168466 (PMC5179079; doi:10.1371/journal.pone.0168466)

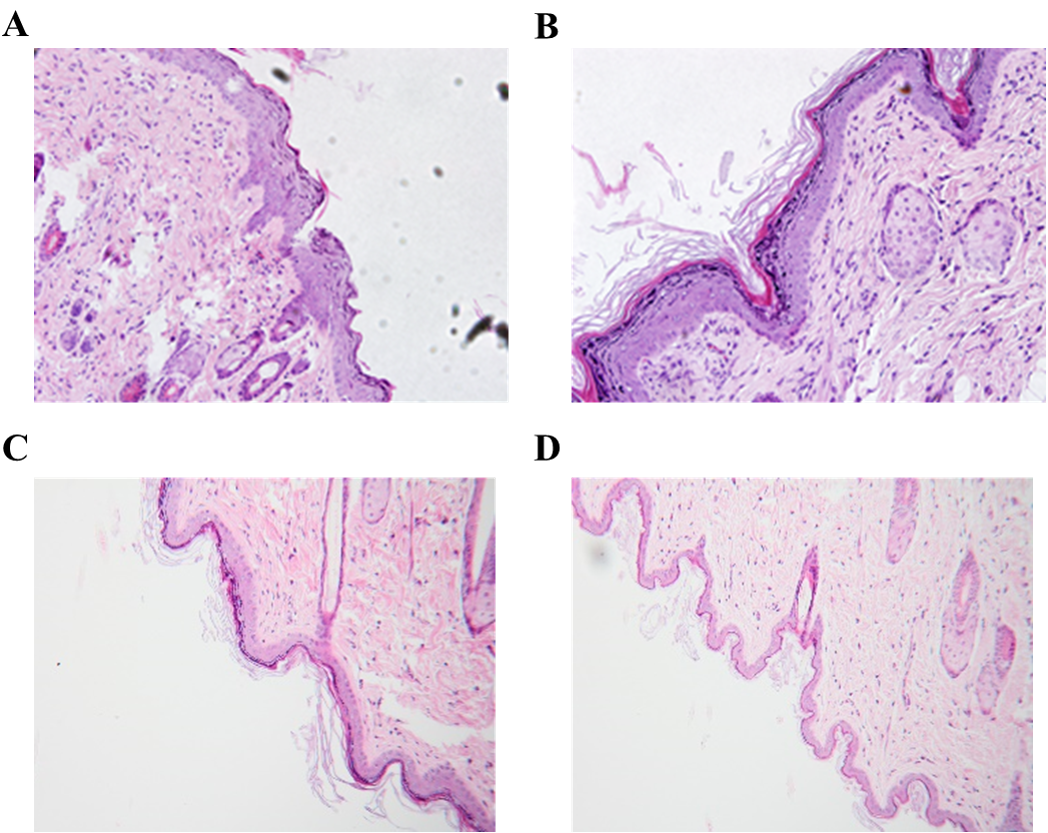

Supplement: S1 Fig — No significant difference was observed in representative presentation from the skin lesion of 1.2FA-AD rat (A) and the Air-AD rat (B). No significant alteration was observed in representative presentation from the skin lesion of 1.2FA-naive rat (C) and the Air-naive rat (D). (TIF) [file pone.0168466.s003.tif]
